# Farmers’ perceptions and acceptance of crossbred guinea pigs in Mantaro Valley, Peru

**DOI:** 10.1007/s11250-023-03465-y

**Published:** 2023-02-10

**Authors:** J. I. Cedano-Castro, R. Jiménez, A. Huamán, G. Gutiérrez Reynoso, M. Wurzinger

**Affiliations:** 1 Universidad Nacional Agraria La Molina, Lima, Peru; 2 Universidad Privada Antenor Orrego, Trujillo, Peru; 3 Instituto Veterinario de Investigaciones Tropicales Y de Altura, Universidad Nacional Mayor de San Marcos, El Mantaro, Peru; 4 University of Natural Resources and Life Sciences, Vienna, Austria

**Keywords:** Breeding strategy, Innovation, Technology transfer

## Abstract

This study aimed at assessing farmers’ perceptions and acceptance of the proposed breeding strategy of Instituto Veterinario de Investigaciones Tropicales y de Altura (IVITA), located in the Central Andes of Peru. A total of 34 farmers, who had received guinea pigs from IVITA, were interviewed. The questionnaire was performed in Spanish language and covered topics about the guinea pig production, feeding, mating system, training, the main products, perception and acceptance of crossbred lines (“cuy RG”), and the farmers’ comment about the terminal crossbred animal (“cuy G”). The preferred feeding strategy was a combination of forage and supplement. All farmers implemented a controlled mating system. Most farmers had received training on feeding, reproduction, management, equipment, and facilities. The main product is animals for slaughter. The 82.40% of farmers reported that they do not purchase guinea pigs from IVITA anymore, because they are highly susceptibility to lymphadenitis, which is a highly contagious disease and causes swollen lymph nodes and affects the meat quality. Most of the respondents classified the “cuy RG” and “cuy G” as suitable for meat production and were satisfied with the performance of the animals. Therefore, 58.80% of respondents already recommended “cuy RG” to other farmers. However, 17.70% of the respondents would not recommend these animals because they are susceptible to disease, especially lymphadenitis. Therefore, IVITA should engage in more structured dialogue with farmers and regularly include their opinion in future improvement of its breeding strategies. This could help to reach higher acceptance rates by farmers.

## Introduction

In Peru, guinea pigs were traditionally raised for home consumption and therefore contributed to rural households’ food security in the Andean region (Chauca 1997). However, in the last decades, guinea pig production turned from a subsistence activity into a profitable business in the highlands and the coastal region of Peru (Chauca 2013; Avilés et al. 2014). The last official data on guinea pig production dates back to 2017. This year, a total number of 827,234 farmers produced 21,103 tons of meat (INEI 2017).

Different authors (Chauca 1997; Lammers et al. 2009; Avilés et al. 2014) describe three production systems for guinea pigs. The first one is called “family system” (around 20 guinea pigs), which is typically found in the highlands, and its purpose is producing meat for home consumption. Farmers use local genotypes of guinea pigs, which are kept inside the kitchen near the stove or fire and fed with organic waste and forage. “Family commercial” (about 100 to 500 guinea pigs) and “commercial systems” (more than 500 guinea pigs) are developed close to urban areas in the highlands but also in the coastal regions. Both systems are characterized by keeping highly specialized purebred or crossbred guinea pigs for meat production. Animals are kept in stables, where they are provided with forage and concentrate. The production is for the local market and export, mainly to the USA.

Over the last decades, the National Institute for Agricultural Innovation-INIA (Instituto Nacional de Innovación Agraria) has developed different improved genetic lines and breeds such as “Peru,” “Andina,” “Inti,” and “Kuri” to support farmers in the improvement of their production (INIA 2004; 2005; 2021).

**Fig. 1:**
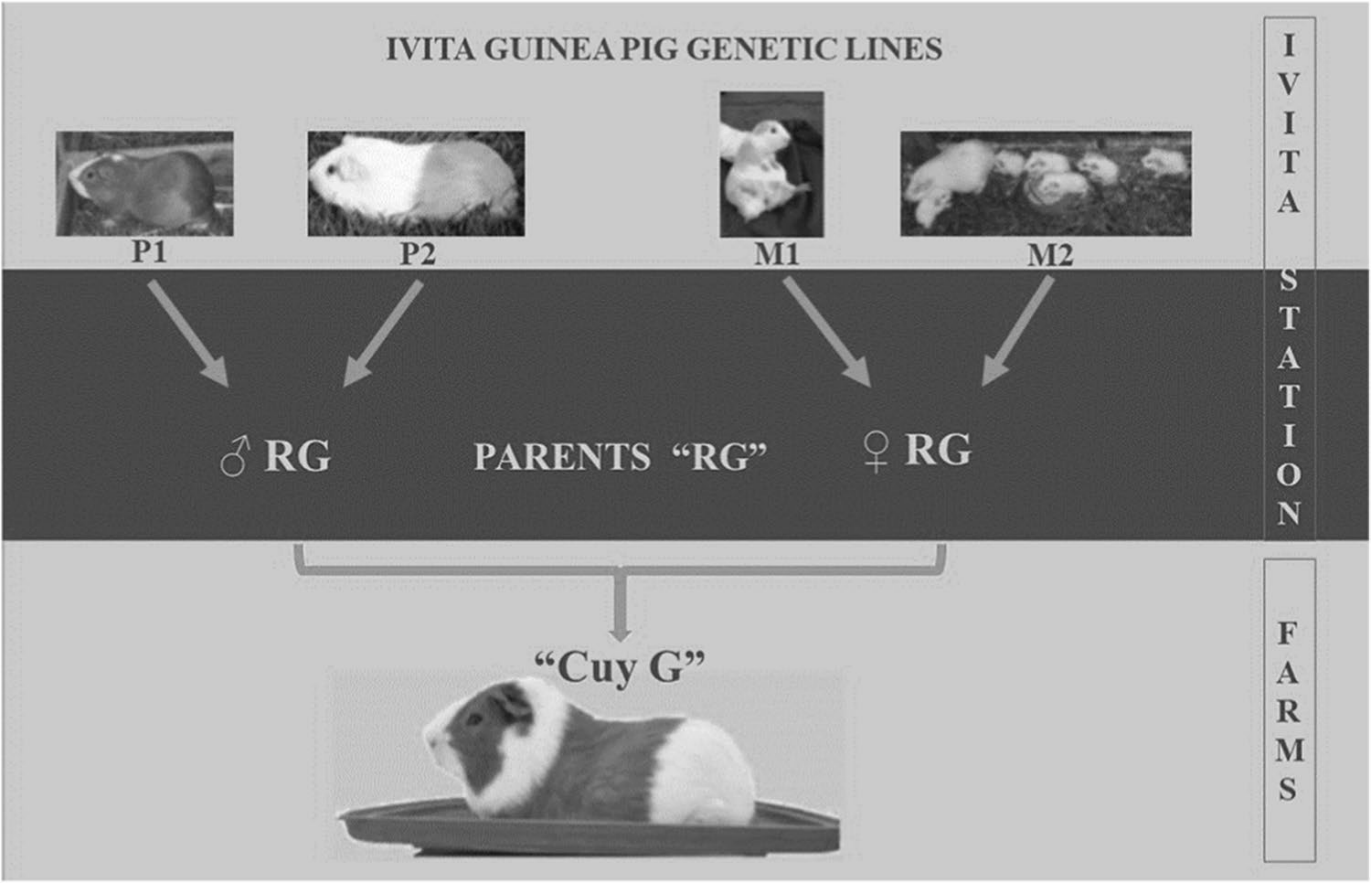
IVITA guinea pigs crossbreeding scheme (adapted from Jiménez and Huamán 2010)

In 2007, the Veterinary Institute for Tropical and High Altitude Research – IVITA (Instituto Veterinario de Investigaciones Tropicales y de Altura) also started by designing a four-line crossbreeding scheme to provide farmers with improved animals (Fig. 1). Two paternal lines, namely, P1, selected for growth rate, and P2, for low feed conversion rate, and two maternal, lines, namely, M1, selected for growth rate of litter, and M2, for high prolificacy (litter size at birth), were developed (Jiménez and Huamán 2010).

IVITA’s breeding program is organized in such a way that F1 crossbred male and female animals (“cuy RG”) are bred at the station. In the next step, farmers purchase both male and female F1 and crossbreed these to get the final marketable product “cuy G.” Besides providing the animals, IVITA offers training to guinea pig farmers about feeding, management, sanitation, and reproduction using attendance-based courses and farm visits and distributing a technical manual (Jiménez and Huamán 2010). With this strategy of a “technology package,” IVITA wants to offer farmers a comprehensive, ready-to-use product.

Through better genetics, feeding, and husbandry measures, productivity should be improved, and income increased. In the literature, however, various factors are mentioned that make technology adoption by farmers difficult or even impossible. Technology uptake is a complex nonlinear process that is influenced by multiple factors. These include knowledge, perception, and farmer’s attitude towards a given technology (Meijer et al. 2015). Farmer’s attitudes are shaped by many factors such as personal characteristics (gender, age, marital status, etc.), socioeconomic characteristics (access to credit, land size, income, assets, education, etc.), personality characteristics (e.g., self-confidence), and familiarity with a technology. The agro-ecological environment, societal, cultural, and political conditions also play a role in the decision-making process. Furthermore, the proposed technology’s characteristics (user-friendliness), benefits, and costs for the farmers’ household economy have to be considered (Meijer et al. 2015; Escobal 2017; Lambrecht et al. 2014).

Furthermore, farmers’ active participation, perceptions, and needs have been discussed as critical elements for breeding programs’ success (Wurzinger et al. 2011; Wurzinger and Gutierrez 2017, Haile et al. 2018).Therefore, this study aimed at assessing farmers’ perception and acceptance of the proposed breeding strategy of IVITA. The study focused on two systems, namely, the family commercial and commercial system.

## Materials and methods

### Study area

**Fig. 2:**
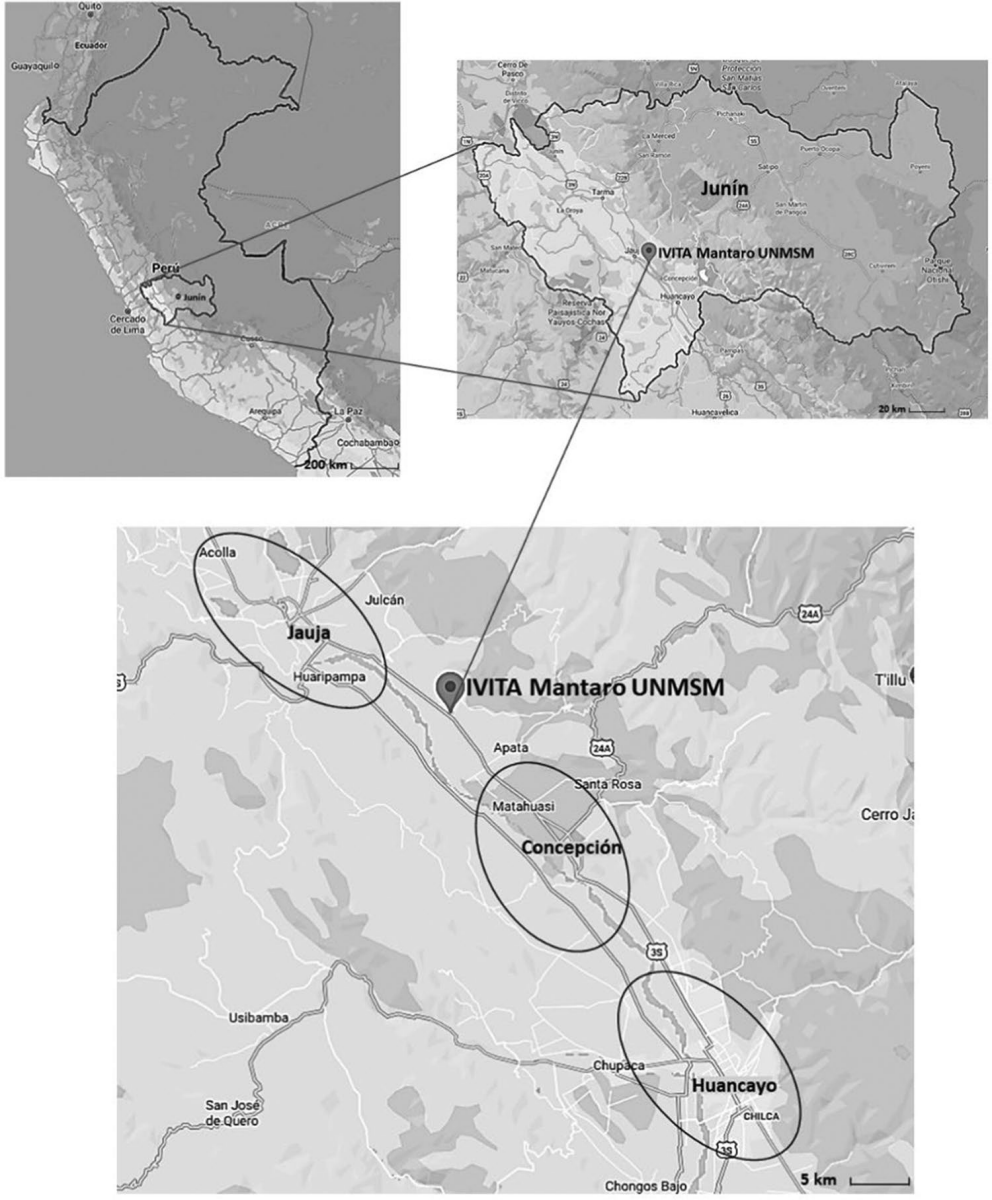
Location of IVITA and the study areas in Mantaro Valley (Jauja, Concepción and Huancayo)

In February 2020, the study was carried out in three provinces of Mantaro Valley (Concepción, Jauja and Huancayo) (Fig. 2), located in the Junín region, Peru. This area’s temperature ranges between 5 and 20 °C during the year and has an annual rainfall of 760 mm (SENAMHI 2020). Mantaro Valley is a productive agricultural area for various crops such as potatoes, maize, wheat, barley, and legumes. Besides, the valley is an area that is also well-known for cattle, sheep, and guinea pig production (Cuellar and Medina 2009).

### Data collection and analysis

Based on IVITA’s sales records between 2016 and 2019, a list of potential interview partners was drawn up. The farmers were contacted by telephone to see if they were interested in joining the study. It was explained to them that participation was voluntary and that their data would be used anonymously. Finally, 34 guinea pig farmers allowed us to visit them. Seventeen farmers were from Concepción (13 family commercial and 4 commercial farms), 12 from Jauja (10 family commercial and 2 commercial farms), and 5 from Huancayo province (all of them of family commercial farms). Figure 2 shows the study region.

Based on Chauca (1997), farms were grouped into family commercial (28 farms) and commercial ones (6 farms). Chauca (1997) uses the number of animals kept as a classification criterion. Farms with 100 to 500 guinea pigs are classified as family commercial ones, and farms with more than 500 guinea pigs are considered commercial farms.

The questionnaire was performed in Spanish language and consisted of open and closed questions. A pre-test of the survey was carried out to ensure that questions are clearly formulated and can be understood by the interviewees. Before starting the interviews, we obtained free prior, informed consent from each respondent and anonymized data before analysis.

The questionnaire covered an extensive range of topics, such as general information about the guinea pig production, feeding, mating system, training, the main products, perception, and acceptance of crossbred guinea pigs (“cuy RG”), and the farmers’ comment about the final crossbred animal “cuy G.” All the information collected was entered into a database.

Data were coded in Microsoft Excel and transferred to Software Package for descriptive analysis.

## Results

### General farm characteristics

Table 1 shows the main characteristics of the studied guinea pig farms. Six commercial and 28 family commercial farms participated in the study. The commercial farms have on average 1318 breeding females, whereas the family commercial farms have only 192 females. It should be noted, however, that the commercial farms show a high standard deviation for the number of breeding females. Respondents have been raising guinea pigs for around 10 years. All farmers of the commercial production system and 64% of the farmers (18 farmers) of the family commercial production system use a mixed diet of forage and a supplement. Of the remaining 10 family commercial farms, 5 farms provide only forage, and 5 farms use forage in combination with commercially produced concentrate.

**Table 1:** Main characteristics of guinea pig farms

Description	Family commercial farm (*n* = 28)	Commercial farm (*n* = 6)
Period of rearing guinea pigs in years (mean ± sd.)	10.5 ± 7	11.5 ± 5
Number of breeding females (mean ± sd.)	192 ± 122	1318 ± 836
Number of breeding males (mean ± sd.)	27.4 ± 24	112 ± 30
Number of total animals (parents + offspring) (mean ± sd.)	423 ± 270	3166 ± 2937
Feeding strategy		
- Only forage	5	-
- Forage + supplement^1^	18	6
- Forage + concentrate^2^	5	-
Mating system		
- Controlled mating	28	6

^1^supplement: mixed of wheat bran, barley, maize, and soy cakes. ^2^concentrate: commercial concentrate

All respondents implemented a so-called controlled mating system. In this system, the breeding females are divided into 3 groups. The breeding males are placed in the 1st group for 3 weeks. After a 1-week recovery period, the males are placed in the 2nd group and then in the 3rd group. In the meantime, the females of the 1st group have finished their pregnancy (around 66 days), and the young animals are weaned. After a week of recovery, the females of group 1 are mated again. This system guarantees the farmer a continuous supply of slaughter animals.

Training is considered essential to improve any business. Almost all farmers (94.10%) have received some training about feeding, reproduction, management, health, equipment, and facilities. Seventy-five percent of the interviewees received training by IVITA, and the remaining 25% received support from other institutions (universities or NGOs).

### Products and market prices

**Fig. 3:**
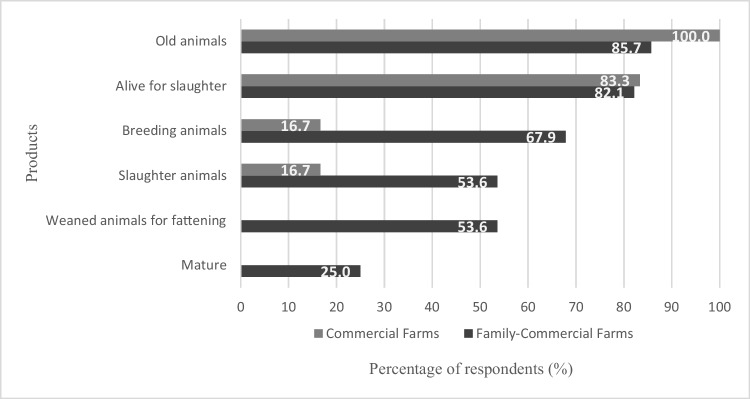
Sales products from guinea pig keeping

Table 2 and Fig. 3 summarize the different products marketed by farmers, their prices in US dollar ($), and local currency (S/, Sol) (base on exchange rate for February 2020) but also the average weight of animals as mentioned by farmers.

**Table 2:** Type, weight, and price of product marketed by farmers in Mantaro Valley

Type of product	Weight of product (g)	Price (US $)^*^ (mean ± sd.)	Local currency (S/) (mean ± sd.)
Old animals^1^	1839 ± 359	6.80 ± 1.31	23.30 ± 4.51
Alive for slaughter	952 ± 131	5.60 ± 0.49	19.30 ± 1.67
Breeding animals	-	8.70 ± 3.46	30.00 ± 11.90
Slaughter animals^2^	846 ± 185	6.20 ± 0.64	21.40 ± 2.19
Weaned animals for fattening	-	4.40 ± 2.05	15.00 ± 7.05
Manure bag (about 25 kg)	-	2.40 ± 1.41	8.10 ± 4.85

^1^breeding animals after 3 or 4 cycles of reproduction. ^2^carcass with the head, legs, liver, lung**,** and kidneys

^*^Price is per animal

The main product offered by farmers is live guinea pigs for slaughter. The 82.00% of the farmers interviewed sold live animals for slaughter at the age of 8 to 12 weeks, with an average weight of 952 g at a price of US $5.60 (Table 2 and Fig. 3). Furthermore, all respondents sold parent guinea pigs after 3 or 4 births (old animals) at US $6.80 with an average live weight of 1839 g. This type of product is mainly sold to restaurants. Other products offered are breeding animals at 15 days of age, slaughtered guinea pigs (about 8 weeks of age and with 846 g), weaned animals (15 days), and manure bags.

### Perception and acceptance of “cuy RG”

Most interviewees (*n* = 28) stated that they no longer purchase animals from IVITA; the remaining six farmers explained that they still purchase occasionally animals (Table 3). The main reason of farmers for this decision is that animals from IVITA (“cuy RG”) are considered highly sensitive to lymphadenitis. This disease causes swollen lymph nodes, which affect the meat quality and makes meat production costly because farmers have to spend money on its treatment. It is also interesting to note that farmers also take their initiative and select the replacement animals from their own herd. Farmers associated this practice with the development of their own genetic line. Seven farmers, whose responses were categorized as “others,” participated in an extension project organized by an NGO, which provided farmers just for one time with animals “cuy RG” and offered specific training. These farmers did not continue with the purchase of breeding stock from IVITA (Table 3).

**Table 3:** Reasons why farmers do not purchase “cuy RG”

Reasons	Family commercial farm		Commercial farm		Total	
	N° of respondents	Percentage (%)	N° of respondents	Percentage (%)	N° of respondents	Percentage (%)
Suffer from lymphadenitis Develop own genetic line High price Purchase from other places IVITA is so far away Others Total	7 5 1 2 2 6 23	30.40 21.70 4.40 8.70 8.70 26.10 100.00	3 1 - - - 1 5	60.00 20.00 - - - 20.00 100.00	10 6 1 2 2 7 28	35.80 21.40 3.60 7.10 7.10 25.00 100.00

### Recommendations for raising “cuy RG”

The package promoted by IVITA consists of the purchase of male and female breeding animals (“cuy RG”) and additional recommendations about improvement in four areas (feeding, mating system, equipment, and facilities) (Jiménez and Huamán 2010). The farmer’s handbook mentions 8 points for improving the stables: protection from predators, protection from pathogens, safe workplace for workers, control of indoor temperature, adapted ventilation and lighting, erection of fences, and the provision of lying areas. For the facilities, feeding troughs, water points (nipple drinkers), and special hiding places for young animals are recommended. However, only 26.50% of respondents followed all areas of recommendations, 14.70% implemented only recommendations of one area (feed, mating system, or equipment), and about one-third did not follow any recommendations (Table 4).

**Table 4:** Recommendations of IVITA followed by the farmers

Recommendation areas followed	Family commercial farm		Commercial farm		Total	
	N° of respondents	Percentage (%)	N° of respondents	Percentage (%)	N° of respondents	Percentage (%)
None Feed Mating system Equipment Feed and facilities Feed and mating system Mating system and facilities Mating system and equipment Feed, mating system and equipment Mating system, equipment, and facilities All four areas Total	10 1 3 1 1 - 2 2 1 - 7 28	35.70 3.60 10.70 3.60 3.60 - 7.10 7.10 3.60 - 25.00 100.00	1 - - - - 1 - 1 - 1 2 6	16.67 - - - - 16.67 - 16.67 - 16.67 33.32 100.00	11 1 3 1 1 1 2 3 1 1 9 34	32.34 2.94 8.82 2.94 2.94 2.94 5.88 8.82 2.94 2.94 26.50 100.00

The key recommendation of IVITA is that farmers purchase the paternal “cuy RG” and the female “cuy RG” and produce a terminal cross “cuy G.” However, only 11.80% (4/34) of respondents have followed this recommendation. Eighteen out of thirty-four farmers explained that they mate “cuy RG” male with female animals from their farms (Table 5).

**Table 5:** Mating of guinea pigs performed on farms

Mating	Family commercial farm		Commercial farm		Total	
	N° of respondents	Percentage (%)	N° of respondents	Percentage (%)	N° of respondents	Percentage (%)
Between “cuy RG” ♂ “cuy RG” with ♀ farm ♂ “cuy RG” with ♀ “cuy RG” and farm ♀ “cuy RG” with ♂ farm Total	3 13 7 5 28	10.70 46.40 25.00 17.90 100.00	1 5 - - 6	16.70 83.30 - - 100.00	4 18 7 5 34	11.80 52.90 20.60 14.70 100.00

### Acceptance of “cuy RG”

Most of the respondents classified “cuy RG” (79.40%) as good parent generation and their offspring “cuy G” (82.40%) and as a good guinea pig for good meat production. They highlighted traits such as large litter size at birth and good maternal abilities for females of the parent generation and robust and heavy males as positive traits. Moreover, farmers described the offspring of “cuy RG” as fast-growing animals. However, seven out of thirty-four farmers classified the parent animals “cuy RG” equally good as their own animals or even with a lower performance. Furthermore, six out of thirty-four farmers had the same opinion about the offspring “cuy G.”

Further, 58.80% of respondents said they had already recommended “cuy RG” as parent generation; 23.50% would probably recommend them. In contrast, 17.70% would not recommend them because of IVITA’s guinea pigs’ perceived susceptibility to disease, especially lymphadenitis.

In Table 6, we summarize traits that farmers would like to be improved in the “cuy RG.” Disease resistance ranked first, followed by changes in body shape and body size.

**Table 6:** Characteristics of “cuy RG” that farmers would like to be improved

Traits to improve	Family commercial farm		Commercial farm		Total	
	N° of respondents	Percentage (%)	N° of respondents	Percentage (%)	N° of respondents	Percentage (%)
Disease resistance Body shape Body size Body shape and disease resistance Body shape and body size Disease resistance and body size All three traits Nothing Total	14 6 1 2 1 1 1 2 28	50.00 21.40 3.60 7.10 3.60 3.60 3.60 7.10 100.00	2 3 - - - 1 - - 6	33.30 50.00 - - - 16.70 - - 100.00	16 9 1 2 1 2 1 2 34	47.07 26.47 2.94 5.88 2.94 5.88 2.94 5.88 100.00

## Discussion

### General farm characteristics

Herd size is an important differentiator between farms in the two production systems. A large proportion of interviewees (100% of commercial and 64% of family commercial farmers) feed guinea pigs with mixed diet of forage and a supplement, which consists of wheat bran, barley, maize, and soy cakes. Respondents mentioned that supplementation covers around 30% of the diet. This result shows that most farmers interviewed were trying to provide their animals with an adequate feed. This feeding strategy was already recommended by some authors (Reynaga 2018; Solorzano 2014). This also means that farmers are investing money for covering the feed costs, which represent around 44% of total cost or 60% of the fixed cost (Herrera 2016; Pascual et al. 2017). This represents a limitation for farmers and could explain the reason why not all farmers in family commercial.

### Products and market prices

In market research carried out by Ordóñez (2003), the marketable weight and age for slaughter animals was 800 g at 80 to 90 days and 1000 to 2000 g for old animals. In our study, farmers mentioned similar weights for their slaughter animals. In contrast, other authors (Chauca 2013; Chauca et al. 2004) recommend targeting animals with 1000 g at 8 weeks of age. This faster growth can only be achieved by more intensive feeding schemes. Guinea pig meat is often consumed in special events such as anniversaries, birthdays, or fairs. This leads to higher market prices than for other commonly consumed meat types such as chicken, beef, or pork, which are sold for US $1.52, US $2.53, and US $2.91, per kilogram, respectively (INEI 2020).

The high price for guinea pigs is reflected in a lower consumption of this meat type. In Peru, the annual per capita consumption of guinea pigs was stated as 0.66 kg in 2016, whereas per capita consumption for chicken, beef, and pork meat was 50.30, 6.10, and 5.40 kg, respectively, in 2018 (MINAGRI 2019; 2020). However, the actual consumption of guinea pig meat might be underestimated as home consumption and informal markets are not reflected in the statistics.

### Perception and acceptance of “cuy RG”

#### Purchase of “cuy RG”

Only the 18% of interviewees continued to purchase animals from IVITA. Farmers lost interest about IVITA’s guinea pigs, because “cuy RG” is in their experience highly sensitive to lymphadenitis, a highly contagious infectious disease for guinea pigs. It is caused by *Streptococcus pyogenes* and *Streptobacillus* and causes swollen lymph nodes and affects the meat quality (Chauca 1997; Estupiñán et al. 2018). According to Jiménez (2016) and Jiménez and Huamán (2010), this disease does not cause death in guinea pigs but makes meat production costly as farmers have to spend money on its treatment or removed all infected animals from the farm. However, there is no literature available if different guinea pig lines are more or less susceptible for this disease. Therefore, it is crucial to ensure that farmers are offered healthy animals; otherwise, they are searching for an alternative source of breeding animals.

Our study shows that farmers follow different breeding strategies. The most common strategy is to buy male breeding animals and mate them with their own female offspring. In contrast, the recommendation of IVITA to purchase both male and female animals is only implemented by four farmers. Purchasing only male breeding stock makes farmers less dependent on constant purchases from an outside source and can minimize the risk of introducing diseases into their herd. Therefore, the proposed 4-line crossbreeding strategy, where farmers have to purchase constantly breeding stock from IVITA, seems not to align with their production strategy.

#### Recommendations for raising “cuy RG”

IVITA’s package involves recommendations of different aspects of guinea pig production. The recommendation of a “controlled mating system” is followed by all farmers by organizing the female population into three groups and mate each group every 30 days. This mating system increase females’ fertility and body weight and the survival rate of offspring at weaning and increases also litter size (Velásquez et al. 2017). In contrast to this, Chauca (1997) suggested implementing continuous mating to take advantage of postpartum heat. However, a controlled mating system allows planning a monthly meat production and all activities on the farms (Jiménez and Huamán 2010; Velásquez et al. 2017).

The respondents explained that IVITA recommends a combination of forage and a mix of grains as a supplement. The supplement should be made of wheat bran, maize, and soy cakes (3–2–1 proportion) and cover around 30% of the diet to provide enough protein and energy and ensure reasonable growth rates (Jiménez and Huamán 2010). Nevertheless, without feed analysis, it is impossible to know whether this supplement has about 15 to 18% protein and 2.8 to 3.0 Mcal of digestible energy /kg as is suggested for this species (Carbajal 2015). This feeding strategy improves the females’ fertility, reduce the mortality rate, and increase the weight gain of growing animals. Moreover, the supplement helps to cover the forage scarcity in the dry season. Thus, the supplement’s use can help improve production efficiency of farm (Jiménez 2007; Velásquez et al. 2017).

Following the IVITA’s recommendation in terms of equipment, 8 respondents implemented nipple drinkers to supply water inside the cages and 7 persons installed feeding troughs. The nipple drinkers help to reduce the waste of water and makes the cleaning of the cages easier. In terms of facilities, farmers replaced the soil floor with a concrete floor in the cages and increased natural lighting areas. These recommendations are in line with Chauca (1997), who mentioned that facilities have to be easy to clean, have enough natural lighting areas, and good ventilation to satisfy the species’ requirement. The fully equipped stable should also allow the management of the microclimate inside through the control of the temperature, humidity, and ventilation (Mosqueira 2019).

There are few reports of experimental studies about using technologies on guinea pig farms like automatic temperature and humidity control or cage-housing systems (Arias and Araujo 2013; Huamaní 2017). The authors explain that the implementation of these technologies would reduce the workload for farmers.

The reasons mentioned by interviewees for not implementing IVITA’s recommendations so far are spending more money, labor, and time. At the same time, two-thirds of the farmers indicated that they have already implemented this sort of recommendation and did not see any need for further improvements of their facilities. Difficult access to loans and the time limitations by farmers have been reported as limiting factors in technology adoption (Meijer et al. 2015; Escobal 2017; Lambrecht et al. 2014). However, it is essential to consider that investments in improving facilities and new equipment could be beneficial in the long term and lead to improved production.

Farmers apply only partly IVITA’s recommendation about mating as they can only afford to buy regularly new breeding males, but this might lead to a lower increase in productivity. In addition, farmers often consider that genetic improvement is carried out by introducing new male animals and do not consider it relevant to also improve the female animals. One option could be to occasionally purchase females “cuy RG” and switch between IVITA and their own females. In both breeding strategies (male “cuy RG” × female “cuy RG” OR male “cuy RG” × own females), a positive heterosis effect can be expected but may be smaller in magnitude in the second option. For farmers with limited economic resources, this could be a viable breeding strategy as also suggested by Blasco and Gou (1992) for pig farmers. This strategy would increase reproductive and productive traits (e.g., litter size and weight gain) and improve adaptation to different environments (Blasco and Gou 1992). This can be translated into more meat produced in less time, and therefore, more income could be generated.

#### Acceptance of “cuy RG”

In the interviews, the farmers emphasized the good reproductive and production performance of “cuy RG” and their offspring as positive characteristics. The characteristics highlighted by Jiménez and Huamán (2010), such as low feed conversion and high survival rate, however, were not mentioned by the farmers.

Moreover, large proportion of respondents recommend “cuy RG”; however, we should take with care this result due to farmers do not keep any records about the performance of animals, and it only reflects the perceptions of farmers but is not supported by any data.

According to Morales and Barrios (2017), guinea pigs and mice are susceptible to the *Streptococcus pyogenes* and *Streptobacillus*, which cause lymphadenitis. In this context, it has to be considered that there is an antagonistic phenotypic relationship between health or fitness traits and production traits (Emanuelson 1988). Therefore, it could be that the stronger selection for production traits at IVITA leads to more susceptible animals. This point has to be taken into consideration while further developing the breeding lines. As a result, farmers prefer to search for alternative sources of breeding stock or select replacement animals from their own farms. However, there are not many research station or universities which could provide breeding stock to the farmers based on performance or genetic evaluation.

Replacement within the own herd might lead to higher inbreeding levels and related problems (Blasco 2021 ). Therefore, IVITA should react to farmers’ demand and include in its breeding program disease resistance (mainly lymphadenitis) as a breeding objective. This strategy could ensure that healthy animals can be offered to farmers.

According to Chauca (1997), a guinea pig should be large with a wide body and short straight hair close to the body and easy-going temperament. The idea of a guinea pig with those linear traits is deep-rooted in the farmer’s mind because those traits have been related to the critical traits of meat animals for a long time. In the survey, body size and shape were mentioned by farmers as characteristics to be improved, as the current IVITA animals do not meet their expectations.

Farmers see IVITA’s guinea pigs (“cuy RG”) as potential good animals for meat production, but they are asking for improvement of some specific traits. They are especially interested in getting more disease-resistant animals as this is an economic factor for them. These results indicate the importance of a close relationship between research institutions and farmers in designing a breeding strategy. Farmers’ breeding objectives, needs, and production systems should be considered at the initial stage of a breeding program (Wurzinger et al. 2011; Wurzinger and Gutierrez 2017). Therefore, IVITA’s breeding program has to achieve the right balance between improving the economically important traits (litter size, weight gain, or feed conversion rate) and including other traits such as body shape and size and disease resistance.

IVITA’s approach of providing farmers with a “technology package” consisting of improved genetics and management recommendations is partly working. To reach a better acceptance by farmers, IVITA should engage in more structured dialogue with farmers and regularly include their opinion in improving their breeding strategies. The topic of susceptibility to lymphadenitis seemed to be of high priority for farmers. This problem may not only be an issue for breeding, but also an important field of work for vaccine development.

Such farmers–researchers’ cooperation could achieve a higher impact of IVITA’s work in the region in the long run.

## Data Availability

The datasets generated during and analyzed during the current study are available from the corresponding author on reasonable request.
